# Testosterone is protective against impaired glucose metabolism in male intrauterine growth-restricted offspring

**DOI:** 10.1371/journal.pone.0187843

**Published:** 2017-11-16

**Authors:** Suttira Intapad, John Henry Dasinger, Joel M. Fahling, Miles A. Backstrom, Barbara T. Alexander

**Affiliations:** 1 Department of Pharmacology, Tulane University School of Medicine, New Orleans, LA, United States of America; 2 Department of Physiology and Biophysics, University of Mississippi Medical Center, Jackson, MS, United States of America; University of Southampton, UNITED KINGDOM

## Abstract

Placental insufficiency alters the intrauterine environment leading to increased risk for chronic disease including impaired glucose metabolism in low birth weight infants. Using a rat model of low birth weight, we previously reported that placental insufficiency induces a significant increase in circulating testosterone in male intrauterine growth-restricted offspring (mIUGR) in early adulthood that is lost by 12 months of age. Numerous studies indicate testosterone has a positive effect on glucose metabolism in men. Female growth-restricted littermates exhibit glucose intolerance at 6 months of age. Thus, the aim of this paper was to determine whether mIUGR develop impaired glucose metabolism, and whether a decrease in elevated testosterone levels plays a role in its onset. Male growth-restricted offspring were studied at 6 and 12 months of age. No impairment in glucose tolerance was observed at 6 months of age when mIUGR exhibited a 2-fold higher testosterone level compared to age-matched control. Fasting blood glucose was significantly higher and glucose tolerance was impaired with a significant decrease in circulating testosterone in mIUGR at 12 compared with 6 months of age. Castration did not additionally impair fasting blood glucose or glucose tolerance in mIUGR at 12 months of age, but fasting blood glucose was significantly elevated in castrated controls. Restoration of elevated testosterone levels significantly reduced fasting blood glucose and improved glucose tolerance in mIUGR. Thus, our findings suggest that the endogenous increase in circulating testosterone in mIUGR is protective against impaired glucose homeostasis.

## Introduction

Diabetes is increasing worldwide [1] and is a known risk factor for cardiovascular disease, the leading cause of mortality in the U.S. Low testosterone is associated with a greater risk for type 2 diabetes (**T2D**) in men [2,3,4,5]. A high endogenous level of free or total testosterone is associated with a lower fasting blood glucose and prevalence of T2D in men [6,7]. Collectively, these studies indicate that testosterone deficiency in men is a risk factor for diabetes. Yet, whether testosterone replacement has a beneficial effect on glucose metabolism in men is controversial [8,9,10,11].

Low birth weight is associated with an increased risk of T2D and cardiovascular disease [12, 13] implicating the role of early life events in later chronic health. A few studies have examined the sex-specific association between low birth weight and diabetes. Mogren et al. demonstrated that fasting blood glucose levels are elevated in low birth weight women, but not low birth weight men in middle age [14]. A similar finding has been reported by Jornyvaz et al. using the CoLaus prospective population-based study [13]. Using the population based AusDiab Study, Al Salmi showed that the effect of birth weight on the development of diabetes is observed in both men and women although the population-attributable risk percentage is greater for low birth weight women than low birth weight men [15]. Other studies by Zimmerman and colleagues, and by Yarmolinsky et al. also report a greater prevalence of diabetes in low birth weight women relative to low birth weight men [7,16], but low birth weight in men is associated with an increased risk for T2D in later life [17,18].

Using a model of low birth weight, i.e. intrauterine growth restriction (**IUGR**) induced via placental insufficiency in the rat, we previously reported that female growth-restricted offspring develop glucose intolerance as early as 6 months of age, prior to the development of increased adiposity that occurs by 12 months of age [19]. Although blood pressure is not elevated in female growth-restricted offspring by 6 months of age [20], male growth-restricted offspring (mIUGR) demonstrate a significant increase in blood pressure in early adulthood [21] suggesting a sex- and age-specific difference in the developmental origins of cardiovascular risk. Hypertension in mIUGR in young adulthood is associated with an increase in circulating testosterone relative to age-matched male controls [22]. Castration abolishes the increase in blood pressure in mIUGR [22] indicating the permissive role of testosterone in the etiology of increased cardiovascular risk originating in early life. However, in later life testosterone associated blood pressure does not remain elevated in mIUGR relative to their normal birth weight counterparts [23]. Whether IUGR programs impaired glucose homeostasis in mIUGR, and whether a decrease in endogenous testosterone plays a role in the development of this impairment are unknown. Thus, we tested whether mIUGR exhibit delayed development of impaired glucose metabolism. Furthermore, we tested whether age-related decline in circulating testosterone contributes to the development of impaired glucose tolerance in mIUGR.

## Materials and methods

### Animals

All experimental procedures were approved by the Animal Care and Use Committee at the University of Mississippi Medical Center and in accordance with the National Institutes of Health. Rats were housed in a temperature-controlled, 23°C, room with a 12:12-hour light/dark cycle with food and water available ad libitum. Timed-pregnant, Sprague Dawley (**SD**) rats purchased from Harlan Inc (Indianapolis, IN) underwent the reduced uterine perfusion procedure or a sham procedure at day 14 of pregnancy as described below. All dams were allowed to deliver at term with birth weight recorded within 12 hours of delivery. At 48 hours of age, offspring in the control and reduced uterine perfusion litters were culled to 8 pups per dam to ensure equal nutrient access for all offspring. The ratio of male to female offspring remained equivalent after culling when possible; however, only male offspring were used in this study. Offspring were weighed three times weekly until weaning at 3 weeks of age; after weaning, rats were weighed every two weeks. Offspring from 11 pregnant controls and 12 reduced uterine perfusion pregnant dams were randomly assigned to groups studied at 6 or 12 months. Only one male offspring per litter was used per study group. The experimental protocol included control groups subjected to measurement of body composition, fasting blood glucose and glucose tolerance at 6 (N = 8 Control and N = 8 IUGR) or 12 (N = 9 Control and N = 8 IUGR) months of age, and experimental groups: 1) sham operation (intact) (N = 6 Control and N = 6 IUGR), 2) castration (**CTX**) (N = 6 Control and N = 4 IUGR) with measurement of body composition before and after sham, or 3) castration with insertion of a testosterone pellet (T) (N = 7 Control and N = 6 IUGR) followed by measurement of fasting blood glucose and glucose tolerance 6 weeks later. All rats undergoing surgical procedures were anesthetized with 2% isoflurane. At the end of the study, animals were euthanized by exposure to 5% isoflurane following with removal of the hearts.

### Reduced uterine perfusion in the pregnant rat

Reduced uteroplacental perfusion was used to induce IUGR at day 14 of gestation, as previously described [21]. Briefly, a silver clip was placed around the lower abdominal aorta (0.203-mm ID) above the iliac bifurcation and at each branch of the ovarian artery (0.100-mm ID); the sham procedure involved visualization of the uterine horn (control).

### Castration in male offspring

At 11 months of age, animals were randomly selected to undergoes sham or castrated surgeries. As previously described [22], a small median incision was made at the distal tip of the scrotum. Subcutaneous connective tissue was cleared, and the testes were visualized. The muscular sac of the testes was excised and exposed by gently pulling on the cauda epididymis. The blood vessels were tied and the vas deferens with the testes was removed. Muscle and the skin were closed with a suture. The sham operation involved exposure of the testes without isolation.

### Testosterone replacement

The testosterone pellet was implanted subcutaneously on the back of the animal at the same time of castration. Testosterone minipellets (25 mg for 60-day release) were used for continuous release of hormone (Innovative Research of America) at a dose chosen to elevate the circulating levels of testosterone to that observed in young adult mIUGR [22].

### Body composition and fatty liver

Total fat mass and total lean mass were determined in conscious animals using an Echo-MRI-700 (Echo Medical Systems, Houston TX); total liver fat mass was determined after harvest. Results are expressed as gram weight.

### Hormone levels

Circulating testosterone and insulin levels were determined using commercially available kits (Ultra-Sensitive Testosterone RIA kit, Linco Research, St. Charles, MO; Insulin Ultra-Sensitive Rat Insulin ELISA kit, Crystal Chem Inc., Downers Grove, IL).

### Glucose tolerance test (OGTT) and glucose stimulated insulin release in response to glucose challenge

As previously described, animals were fasted overnight before the OGTT [19]. A bolus of D-glucose solution (5 g per kg BW) was administrated orally by gavage. Blood glucose levels were determined from samples collected from the tail vein (FreeStyle Lite glucometer, Abbott Diabetes Care, Alameda, CA) at 0, 30, 60, and 120 min post-gavage. Collected blood samples were also used to determine circulating insulin levels.

### Statistics

Data are presented as mean values ± SEM with n representing the number of male offspring representing one pup per litter per parameter. Graphpad PRISM version 4 (Graph Pad Software, San Diego, CA) was used for all statistical analysis. Differences between groups were evaluated by two-way analysis of variance (ANOVA) followed by Bonferroni posttest with IUGR and age or CTX as sources of variation as applicable. An unpaired student t-test was used for comparison of birth weight and pre- versus post-body composition analysis. Differences were considered statistically significant at *P* < 0.05.

## Results

### Intrauterine growth restriction, testosterone and body weight

**Fig 1:**
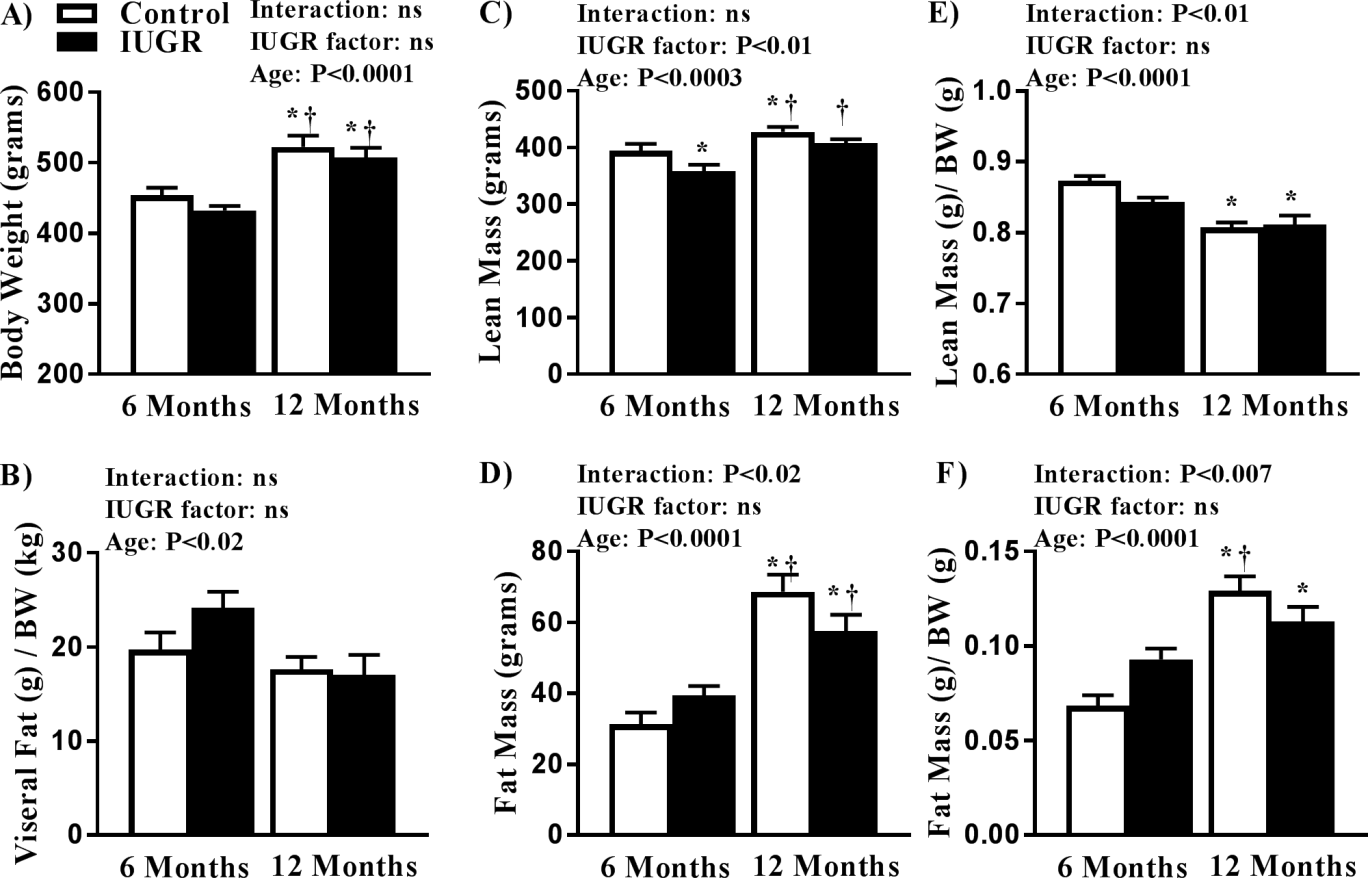
Body weight (A), visceral fat mass (B), total lean mass (C), total fat mass (D), lean mass per gram body weight (E), and fat mass per gram body weight (F) in male control and male growth-restricted (**IUGR**) offspring at 6 or 12 months of age. *P<0.05 versus 6-month control. † *P*<0.05 versus 6-month growth-restricted. Data values represent mean±SE.

Birth weight was significantly reduced in mIUGR compared with male controls (5.01 ± 0.09 vs. 6.17 ± 0.12 grams, *P* < 0.01). Body weight of mIUGR vs. age-matched control offspring did not differ at 6 or at 12 months of age (Fig 1A). Visceral fat was also not significantly different in mIUGR relative to age-matched control at 6 or 12 months of age (Fig 1B). Yet, total lean mass was reduced in relative to control counterparts at 6 months but did not differ by 12 months of age (Fig 1E). Total fat mass was increased in mIUGR and control offspring at 12 months relative to 6 months old groups. (Fig 1D).Lean mass per gram body weight was reduced in mIUGR and control offspring at 12 months old of age relative to control offspring at 6 months (Fig 1E) whereas fat mass per gram body weight was increased (Fig 1F). Castration did not significantly alter body weight, total fat, or total lean mass in control or mIUGR (Table 1). However, total fat mass was reduced in both control and mIUGR administered testosterone following castration (Table 1).

**Table 1:** Body composition in male control and male growth-restricted (IUGR) rats before and 6 weeks after castration (CTX) or castration plus testosterone (T) replacement. Data were analyzed using a two-tailed, paired t-test for comparison of “before and after” in the different groups. Values denote mean ± standard error of the mean. *P<0.05 versus 6 weeks prior to counterpart.

	Control prior to CTX	Control after CTX	IUGR prior to CTX	IUGR after CTX	Control prior to CTX plus T	Control after CTX plus T	IUGR prior to CTX plus T	IUGR after CTX plus T
**Body** **Weight (grams)**	584±19	583±11	565±31	556±28	581±14	575±12	472±15	465±14
**Fat Mass** **(grams)**	94±6	84±7	102±16	100±12	100±7	77±6*	65±7	49±5*
**Lean Mass (grams)**	417±17	378±47	391±17	376±17	390±10	411±29	356±16	334±12

### Intrauterine growth restriction and glucose homeostasis

**Fig 2:**
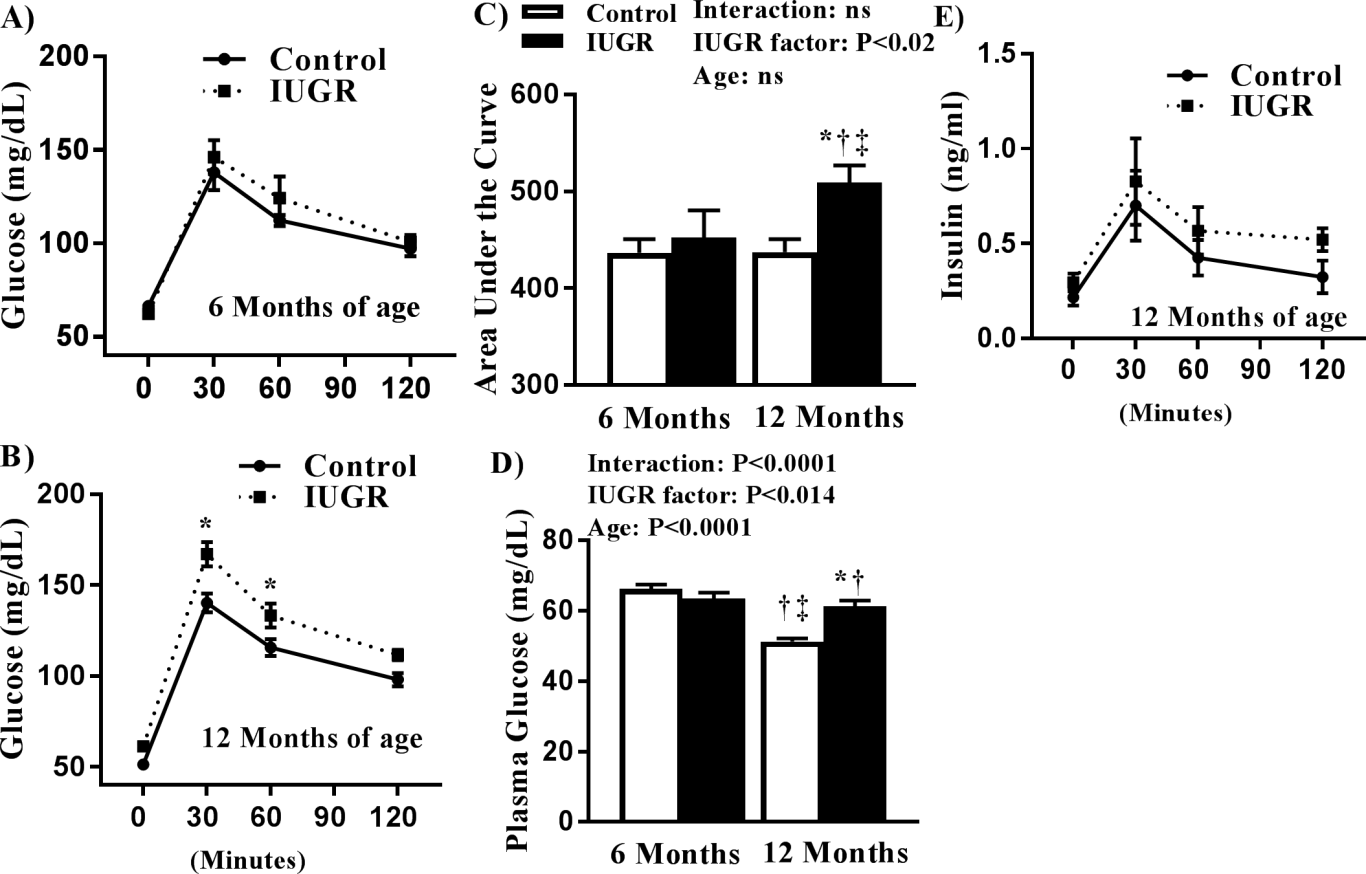
Oral glucose tolerance test (**OGTT**) at 6 (A) and 12 months of age (B) in male control and growth-restricted (**IUGR**) offspring. Area under the curve (**AUC**) (C) and fasting blood glucose (D) at 6 and 12 months of age. The insulin response to a fasting blood glucose challenge at 12 months of age (E). *P<0.05 versus 12-month control. †*P*<0.05 versus 6-month control; ‡ *P*<0.05 versus 6-month growth-restricted. Data values represent mean±SE.

To determine if IUGR programs glucose intolerance in male mIUGR, we examined glucose tolerance at 6 and 12 months of age. The glucose response to a glucose, fasted challenge was not altered in mIUGR relative to 6 month controls (Fig 2A). At 12 months of age, fasted mIUGR exhibited a significant elevation in blood glucose at 30 and 60 min post-glucose challenge compared with age-matched controls (Fig 2B). The area under the curve (AUC) was significantly increased in mIUGR at 12 months of age relative to age-matched controls (Fig 2C). AUC was also significantly increased in mIUGR at 12 months of age compared with mIUGR at 6 months implicating an age effect on glucose homeostasis after mIUGR. No difference was noted in the overnight fasting blood glucose concentration in mIUGR compared with age-matched control offspring at 6 months of age. Overnight fasting blood glucose levels were significantly increased in mIUGR at 12 months of age relative age-matched controls (Fig 2D). Glucose-induced insulin release was not altered post-glucose challenge in mIUGR rats relative to age-matched control at 12 months of age (Fig 2E).

### Testosterone and glucose homeostasis

**Fig 3:**
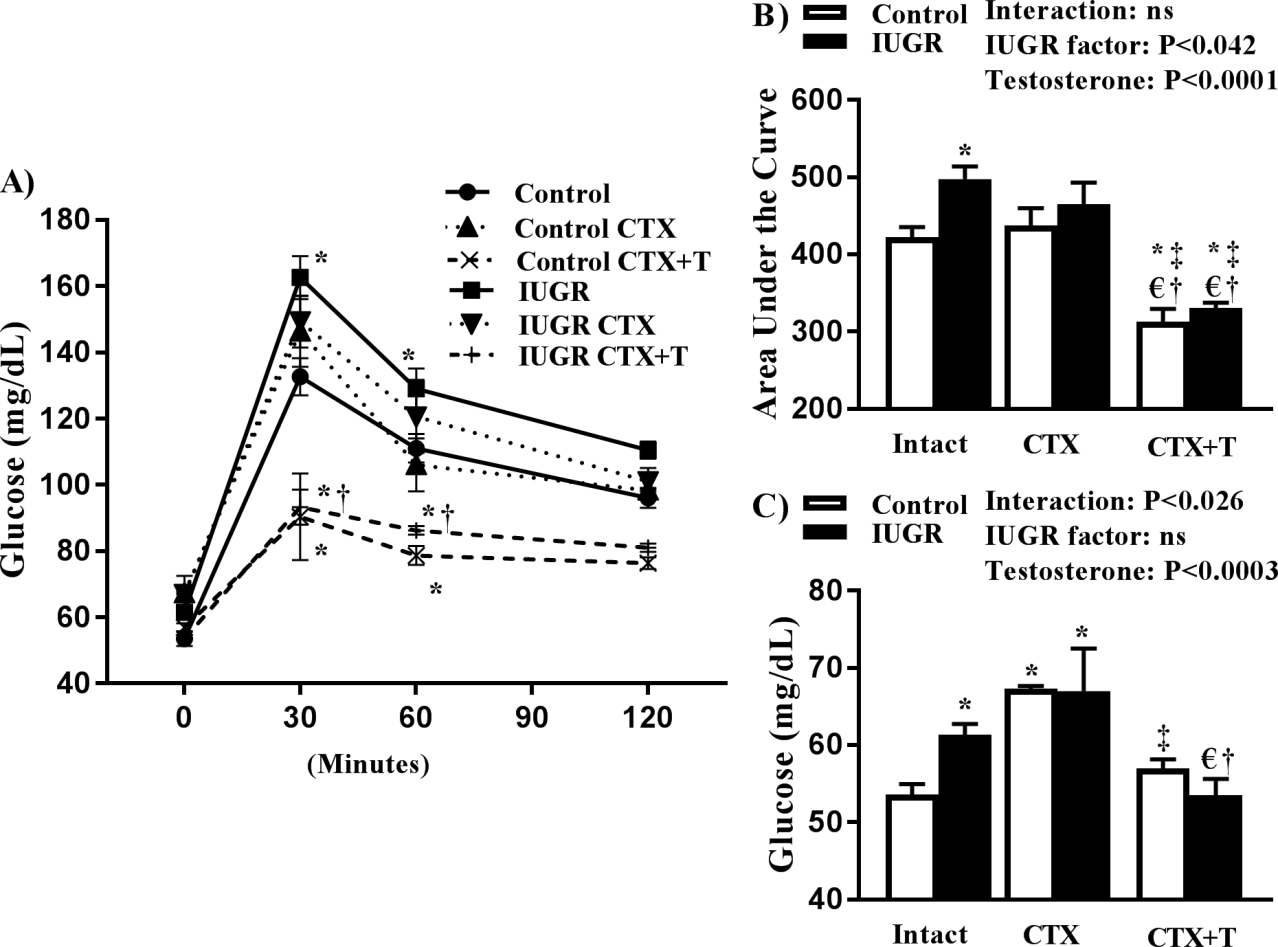
Oral glucose tolerance test (**OGTT**) (A), area under the curve (B), and fasting blood glucose (**AUC**) (C) in intact, castrated, (**CTX**) and castrated plus testosterone replacement in male control and growth-restricted (**IUGR**) offspring. *P<0.05 versus intact control. †*P*<0.05 versus intact growth-restricted. ‡ *P*<0.05 versus castrated control. € *P*<0.05 versus castrated growth-restricted. Data values represent mean±SE.

We previously reported that circulating levels of testosterone are significantly elevated in mIUGR relative to age-matched control in young adulthood [22] but no longer differ by 12 months of age [23]. To determine if changes in testosterone correlate with altered glucose homeostasis, we performed an oral glucose tolerance test 6 weeks after sham, castration, or castration with testosterone replacement. Blood glucose was elevated at 30 and 60 minutes’ post glucose challenge in mIUGR relative to control counterparts (Fig 3A). Castration did not alter the glucose response to a glucose challenge in control or mIUGR relative to their intact counterpart, but castration in conjunction with testosterone replacement significantly reduced circulating glucose levels at 30 and 60 minutes’ post-challenge in control and mIUGR relative to their intact counterpart (Fig 3A). AUC and fasting blood glucose levels were elevated in mIUGR relative to control (Fig 3B, Fig 3C respectively). Castration did not alter AUC or fasting blood glucose in mIUGR relative to intact counterparts, but castration in conjunction with testosterone replacement reduced both of these parameters in mIUGR relative to intact and castrated counterparts (Fig 3B, Fig 3C). Castration increased fasting blood glucose levels, but not AUC in control offspring relative to intact control; testosterone replacement abolished the increase in fasting blood glucose and reduced AUC in control relative to intact and castrated counterparts (Fig 3B, Fig 3C).

### Liver fat mass at 12 months of age, effect of castration and testosterone replacement

Liver fat mass did not significantly differ in in intact mIUGR relative to intact control (9.0 ± 3.6 versus 13.4 ± 2.1 grams and 0.0153±0.006 versus 0.022±0.003 g/gBW mIUGR versus control, respectively) at 12 months of age. Liver fat mass increased significantly in castrated mIUGR and castrated control offspring relative to their intact counterpart (20.5 ± 3.2 and 30.7 ± 6.0 grams; 0.037±0.006 and 0.052±0.010 g/gBW, *P* < 0.05 versus intact counterpart; mIUGR and control, respectively). Liver fat mass was significantly reduced in offspring with chronic testosterone replacement relative to their castrated counterpart (7.0 ± 2.9 and 7.7 ± 3.7 grams; 0.014±0.007 and 0.013±0.002 g/gBW, *P* < 0.05 versus castrated counterpart; mIUGR and control, respectively).

### Circulating testosterone levels in intact and castrated rats

**Fig 4:**
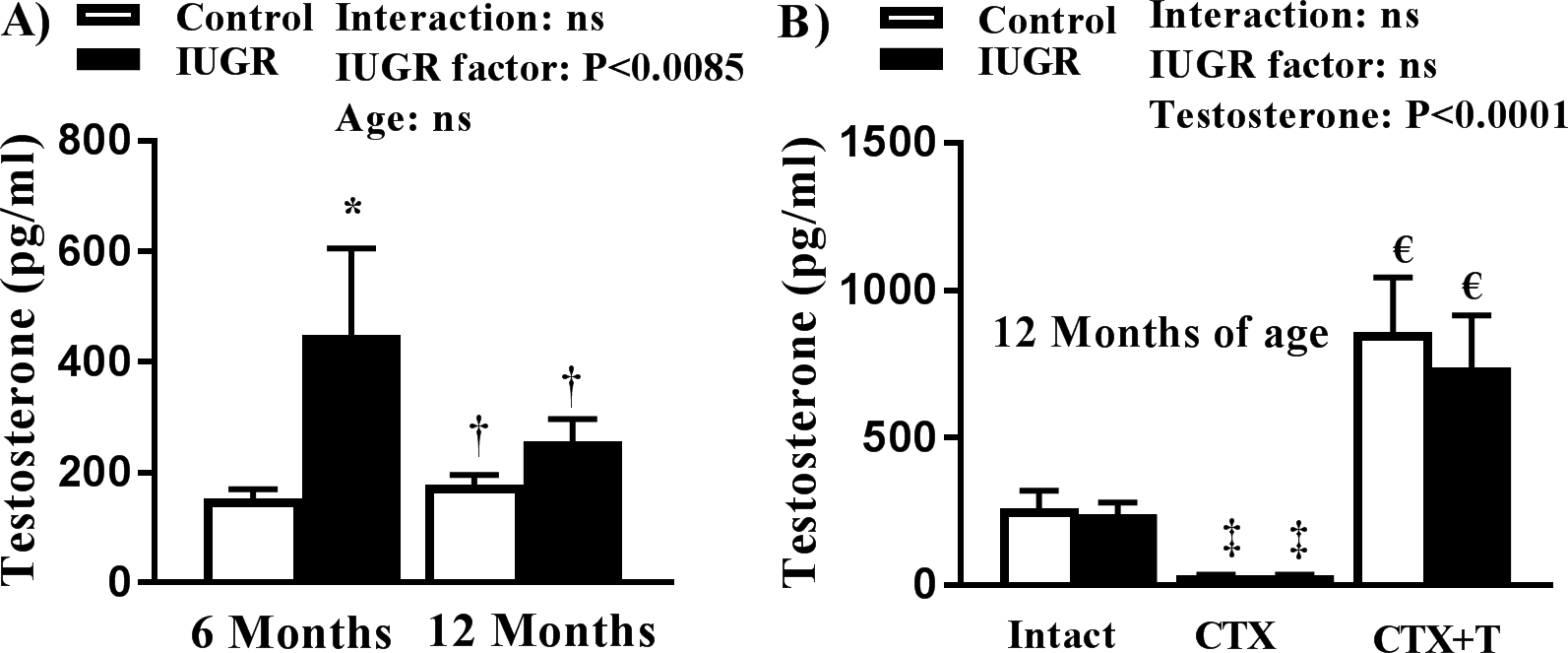
Circulating testosterone levels in male control and male growth-restricted (IUGR) offspring at (A) 6 and 12 months of age, or (B) at 12 months of age in intact, castrated (CTX) or castrated plus hormone replacement (CTX + T). *P<0.05 versus 6-month control. †*P*<0.05 versus 6-month growth-restricted. ‡ *P*<0.05 versus intact groups, € *P*<0.05 versus intact and CTX groups. Data values represent mean±SE.

Circulating testosterone levels were significantly increased at 6 months of age in mIUGR relative to control (Fig 4A). As previously reported [22], circulating testosterone levels were no longer significantly elevated in mIUGR at 12 months of age (Fig 4A, Fig 4B). Castration significantly decreased testosterone levels in male offspring relative to intact contrals (Fig 4B). Testosterone replacement by insertion of a subcutaneous mini-pellet significantly increased circulating levels of testosterone above endogenous levels relative to intact controls (Fig 4B).

## Discussion

This study introduced several new findings. Unlike their female littermates [19], mIUGR did not exhibit glucose intolerance at 6 months of age. However, glucose intolerance was observed at 12 months of age in mIUGR relative to age-matched control. Fasting blood glucose levels were also elevated in mIUGR at 12 months of age relative to age-matched male controls. As previously reported [23], testosterone levels were elevated at 6, but not at 12, months of age in mIUGR relative to age-matched male control. Thus, the development of impaired glucose homeostasis in mIUGR at 12 months of age was associated with normalization of testosterone levels in mIUGR relative to age-matched control. Castration did not alter fasting blood glucose levels in mIUGR, whereas fasting blood glucose levels were elevated in castrated control offspring at 12 months of age. Testosterone replacement elevated levels above intact control normalized AUC and fasting blood glucose in castrated mIUGR at 12 months of age relative to male control counterparts. Taken together, these results indicate that an elevation in endogenous testosterone may be protective against impaired glucose metabolism in mIUGR and that placental insufficiency programs sex- and age-specific alterations in glucose homeostasis in mIUGR.

Different experimental models of developmental insult are used to investigate the mechanisms, which contribute to impaired glucose homeostasis [24,25,26]. Sex differences in glucose metabolism are observed in these models, although findings differ based on the method or timing of the fetal insult. Our current and previous studies from IUGR model induced by reduced uterine perfusion indicate a faster development of impaired glucose homeostasis at a younger age in female IUGR than in male IUGR. Zambrano and colleagues reported that male, but not female, Wistar rat offspring exposed to maternal protein restriction during pregnancy (8 vs. 20% protein) demonstrate an increase in fasting glucose at 110 days of age [26,27]. Fernandez-Twinn et al. demonstrated that female low protein offspring do not exhibit an increase in fasting blood glucose or develop glucose tolerance in later life [28]. However, Petry et al. showed that male Wistar rat offspring exposed to maternal protein restriction (8 vs. 20% protein) exhibit glucose intolerance associated with an increase in fasting blood glucose levels at 17 months of age [29]. Yet, studies by Chamson-Reig and colleagues indicated that female but not male low protein (8 vs. 20%) Wistar rats demonstrate an increase in blood glucose and glucose intolerance in young adulthood [30]. In the rat model of placental insufficiency induced by bilateral uterine ligation (**BUL**) at day 19 of gestation, Simmons showed a significant increase in fasting blood glucose and glucose intolerance due to impaired insulin release in response to a glucose challenge in the fasted, male Sprague Dawley IUGR rat offspring at 6 months of age [25]. However, Siebel et al. reported that male, but not female, Wistar Kyoto rat offspring exposed to BUL at gestation day 18 exhibit impaired glucose metabolism at 6 months of age [31], whereas Jansson and Lambert observed impaired glucose tolerance at 3–4 months of age in female, but not male, Sprague Dawley offspring exposed to unilateral uterine ligation at day 18 of gestation [24]. Taken together, these studies suggest that fetal insult programs sex- and age-specific changes in glucose metabolism in the offspring. However, subtle differences based on the timing or the method of fetal insult, rat strain, or as elegantly reviewed by Jah-Mihan and colleagues, differences in dietary composition can alter programmed outcomes [32]. Simmons reported that BUL during gestation, programs a reduction in beta-cell mass in mIUGR that exhibit fasting hyperglycemia [25]; Chamson-Rieg showed that low protein programs a reduction in pancreatic beta-cell mass associated with impaired glucose tolerance and elevated blood glucose in female low protein offspring in young adulthood [30]. Whether beta-cell mass is reduced in mIUGR in our model is not known. Yet, impaired beta cell function is not always associated with a reduction in beta cell mass following a perinatal insult. Compensatory increases in beta cell mass are observed in young adult male sheep exposed to placental restriction [33]. Differences in pancreatic maturation between species may account for this difference in the developmental programming of altered beta cell mass [34]. However, in contrast to our rodent model of placental insufficiency [19], insulin secretion is impaired in young adult male but not female sheep exposed to placental restriction [35] whereas insulin secretion in our rodent model is blunted only in female IUGR at 12 months of age [19]. Thus, sex-specific programming of beta cell function may also be species specific. Further studies are needed to determine the effect of IUGR induced by reduced uterine perfusion in the rat, in addition to sex, on beta cell mass.

A possible mechanism by which testosterone can alter glucose metabolism may involve changes in body composition. As reviewed by Zerradi et al., androgens modulate body fat distribution via inhibition of adipocyte differentiation [36]. Fat mass was increased in mIUGR at 12 months of age relative to mIUGR at 6 months of age; an increase that corresponded to a decrease in circulating testosterone. Yet, fat mass was also increased in control offspring at 12 months of age relative to control offspring at 6 months of age. Despite differences in fasting blood glucose, the fat mass of mIUGR and control did not differ at 12 months of age. Furthermore, AUC was increased only in mIUGR, but not control offspring at 12 months of age relative to their 6 month counterparts. Testosterone replacement lowered fat mass, AUC, and fasting blood glucose in control and mIUGR implicating that control and mIUGR were both sensitive to the effect of testosterone elevation above endogenous control levels on body composition and glucose homeostasis. In the study by Chamson-Reig, testosterone levels were reduced in male low protein offspring in young adulthood that did not exhibit a change in glucose tolerance or fasting blood glucose levels relative to male control [30]. Adiposity was significantly elevated in conjunction with reduced endogenous testosterone in this study [30]. Whether low protein male offspring in this model of fetal insult develop impaired glucose homeostasis with age is not yet known. Additionally, the exact timing of the age-related decline in endogenous testosterone and the development of impaired glucose homeostasis in our model of mIUGR is unknown. Therefore, whether a reduction in testosterone in mIUGR precedes developmental programming of the impaired glucose homeostasis originating in fetal life has not yet been elucidated.

An increase in endogenous glucose production is the main source of elevated fasting glucose in T2D [10]. An increase in free fatty acids within the liver can increase glucose production within the liver [37]. Thus, an elevation in free fatty acids in the liver with an increase in liver fat deposition can alter metabolism leading to the development of metabolic disease [38]. Testosterone inhibits lipid uptake and stimulates lipolysis[39]. Yet, despite the loss of a high endogenous level of testosterone in mIUGR, fat mass was not elevated in intact mIUGR relative to intact control offspring at 12 months of age. But, castration increased liver fat mass in both mIUGR and control offspring relative to their intact counterpart; this increase was not observed in rats that underwent castration with testosterone replacement. Therefore, an increase in fat mass was associated with an increase in fasting blood glucose in control; an outcome that was prevented by testosterone replacement. An increase in fasting blood glucose was not associated with an increase in liver fat mass in intact mIUGR.

To conclude, our results indicate that a high endogenous testosterone level was protective against the developmental programming of impaired glucose homeostasis in the mIUGR rat. The beneficial effect of testosterone was not linked to an increase in total body fat mass or fatty liver in the intact mIUGR rat. Also, insulin release in response to glucose in the fasted state was not altered. Thus, the mechanism by which elevated endogenous testosterone is protective against impaired glucose homeostasis in the mIUGR rat is unknown. However, we previously reported that elevated endogenous testosterone in the mIUGR rat programs a significant increase in blood pressure in young adulthood [22] and contributes to accelerated cardiovascular aging. [23]. Therefore, the developmental origins of increased endogenous testosterone had an opposite effect on blood pressure and metabolic health of male IUGR.

### Perspectives and significance

Testosterone decreases in aging men [40]. Low testosterone in men is associated with increased risk for T2D [2,3,4,5]. Numerous studies suggest that testosterone replacement in men improves glycemic control [41,42,43,44,45]. Yet, the benefit of testosterone replacement for improved metabolic health in men is controversial [8, 9, 11] because the long-term risk of testosterone replacement in men is linked to increased cardiovascular risk [46,47,48,49]. Thus, the beneficial effect of testosterone replacement on metabolic health must be balanced against the increased risk for cardiovascular events. Further studies are needed to clarify the risk versus benefit of testosterone replacement in men. Moreover, targeted investigations into the mechanisms that contribute to increased risk for impaired glucose homeostasis originating in fetal life are warranted to improve therapeutic and interventional strategies in low birth weight individuals.
